# Crystal structures of two hydrazinecarbo­thio­amide derivatives: (*E*)-*N*-ethyl-2-[(4-oxo-4*H*-chromen-3-yl)methyl­idene]hydrazinecarbo­thio­amide hemi­hydrate and (*E*)-2-[(4-chloro-2*H*-chromen-3-yl)methyl­idene]-*N*-phenyl­hydrazinecarbo­thio­amide

**DOI:** 10.1107/S2056989015003369

**Published:** 2015-02-21

**Authors:** Rajeswari Gangadharan, Jebiti Haribabu, Ramasamy Karvembu, K. Sethusankar

**Affiliations:** a Department of Physics, Ethiraj College for Women (Autonomous), Chennai 600 008, India; b Department of Chemistry, National Institute of Technology, Tiruchirappalli 620 015, India; c Department of Physics, RKM Vivekananda College (Autonomous), Chennai 600 004, India

**Keywords:** crystal structure, hydrazinecarbo­thio­amide, thio­urea derivatives, α-*N*-heterocycle, hydrogen bonding

## Abstract

The title compounds, C_13_H_13_N_3_O_2_S·0.5H_2_O, (I), and C_17_H_14_ClN_3_OS, (II), are hydrazinecarbo­thio­amide derivatives. Compound (I) crystallizes with two independent mol­ecules (*A* and *B*) and a water mol­ecule of crystallization in the asymmetric unit. The chromene moiety is essentially planar in mol­ecules *A* and *B*, with maximum deviations of 0.028 (3) and 0.016 (3) Å, respectively, for the carbonyl C atoms. In (II), the pyran ring of the chromene moiety adopts a screw-boat conformation and the phenyl ring is inclined by 61.18 (9)° to its mean plane. In the crystal of (I), bifurcated N—H⋯O and C—H⋯O hydrogen bonds link the two independent mol­ecules forming *A*–*B* dimers with two *R*_2_^1^(6) ring motifs, and *R*_2_^2^(10) and *R*_2_^2^(14) ring motifs. In addition to these, the water mol­ecule forms tetra­furcated hydrogen bonds which alternately generate *R*_4_^4^(12) and *R*_6_^6^(22) graph-set ring motifs. There are also π–π [inter-centroid distances = 3.5648 (14) and 3.6825 (15) Å] inter­actions present, leading to the formation of columns along the *c*-axis direction. In the crystal of (II), mol­ecules are linked by pairs of N—H⋯S hydrogen bonds, forming inversion dimers with an *R*_2_^2^(8) ring motif. The dimers are linked by C—H⋯π inter­actions, forming ribbons lying parallel to (210).

## Abstract 2

The title compounds, (I) and (II), are hydrazinecarbo­thio­amide derivatives. In the crystal of (I), two independent mol­ecules are linked by bifurcated N—H⋯O and C—H⋯O hydrogen bonds, forming two 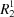
(6) ring motifs, and 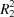
(10) and 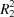
(14) ring motifs. In the crystal of (II), mol­ecules are linked by pairs of N—H⋯S hydrogen bonds, forming inversion dimers with an 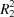
(8) ring motif.

## Chemical context   

Thio­semicarbazones belong to a large group of thio­urea derivatives which are derived from parent aldehydes and ketones. The biological activity of these compounds depends on the parent aldehyde and ketone (Beraldo & Gambino, 2004 ▸). Derivatives of hydrazinecarbo­thio­amide constitute an important group of multidentate ligands with potential binding sites available for a wide variety of metal ions. The chemistry of thio­semicarbazone complexes has received much attention owing to their significant biological activities and medicinal properties. Presently, the areas in which thio­semicarbazones are receiving the most attention are based on their anti­tumour, anti­protozoal, anti­bacterial and anti­viral activities (Finch *et al.*, 1999 ▸; Antholine *et al.*, 1977 ▸). α-*N*-heterocyclic thio­semicarbazones possess anti­tumour properties partially related to their ability to inhibit ribonucleoside reductase (RR), an iron-containing enzyme which is essential in DNA synthesis (Sartorelli *et al.*, 1970 ▸). The medicinal action of these thio­semicarbazones appears to be directly related to their ability to chelate the iron atom of the active site of RR or by destroying the tyrosinase radical present in a subunit of this protein (Thelander & Graslund, 1983 ▸). The structures of the title compounds were determined in order to investigate the extent of electron delocalization, ligand conformations and to illustrate their biological implications.
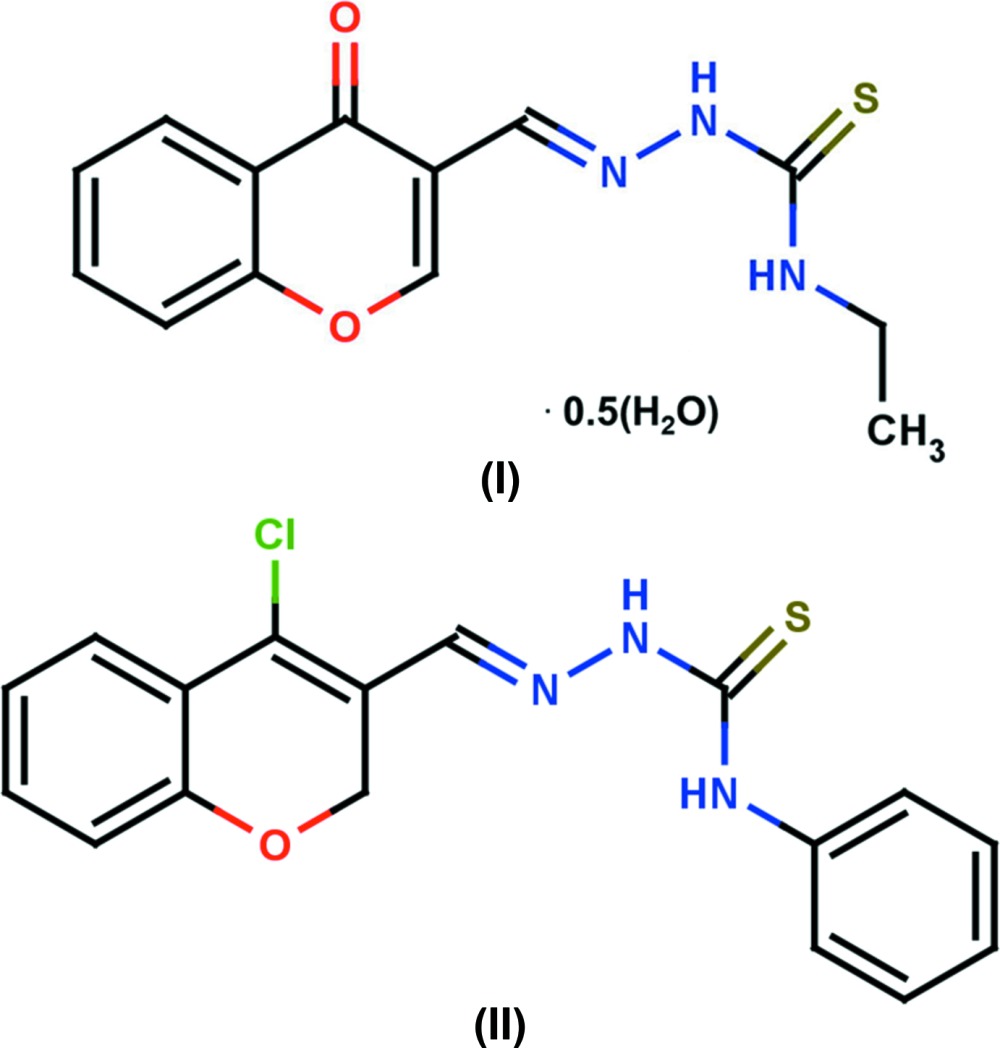


## Structural commentary   

**Figure 1:**
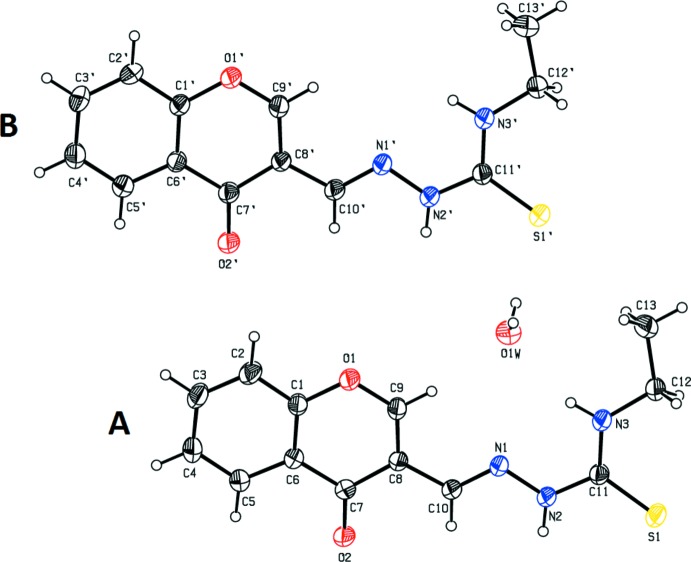
The mol­ecular structure of the two independent mol­ecules (*A* and *B*) of compound (I), showing the atom labelling. Displacement ellipsoids are drawn at the 30% probability level.

**Figure 2:**
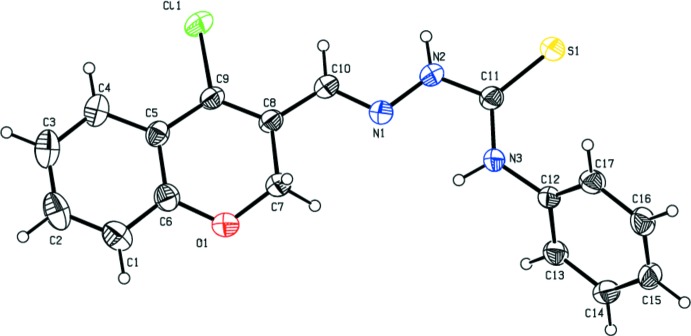
The mol­ecular structure of compound (II), showing the atom labelling. Displacement ellipsoids are drawn at the 30% probability level.

In compound (I) (Fig. 1 ▸), the chromene moieties of mol­ecules *A* and *B* are essentially planar, with maximum deviations of 0.028 (3) and 0.016 (3) Å for atoms C7 and C7′, respectively. However, in compound (II) (Fig. 2 ▸), the chromene moiety is not quite planar with a dihedral angle of 5.67 (12)° between the mean planes of the fused six-membered rings. In compound (II), the pyran ring of the chromene moiety adopts a screw-boat conformation [puckering amplitudes and smallest displacement parameters are *q* = 0.314 (2) Å, θ = 116.4 (4)°, φ = 147.5 (5)° and ΔC_2_ = 0.7 (3)]. In compound (II), the dihedral angle between the chromene moiety and the phenyl ring is 61.18 (9)°. The deviation of the carbonyl O atoms (O2 and O2′) from the mean plane of the pyran ring in compound (I) are 0.0838 (18) and 0.0386 (19) Å in mol­ecules *A* and *B*, respectively, while the deviation of the Cl atom in compound (II) is 0.312 (1) Å.

The hydrazinecarbo­thio­amide backbone is almost planar, with the maximum deviation being exhibited by atom N2 in both compounds; 0.025 (2) and 0.051 (2) Å, respectively, in mol­ecules *A* and *B* of compound (I) and 0.072 (2) Å in compound (II).

Thio­semicarbazones exist in the thione form in the solid state and in solution they exist as an equilibrium mixture of thione and thiol forms (Kurup & Joseph, 2003 ▸). The fact that the compounds exists in the thione form is confirmed by the N—N, N—C and C=S bond lengths.. The C—S bond lengths are 1.681 (2) and 1.673 (2) Å in mol­ecules *A* and *B*, respectively, of compound (I), and 1.668 (2) Å in compound (II). These bond lengths are inter­mediate between normal S—C*sp* ^2^ single-bond and S=C*sp* ^2^ double-bond distances of *ca* 1.75 and 1.59 Å, respectively, indicating the presence of partial double-bond character (Kumbhar *et al.*, 1997 ▸). The N1—N2 bond lengths [varying between 1.367 (2) and 1.369 (2) Å] are very close to that reported for a similar substituted hydrazine­carbo­thio­amide compound (Joseph *et al.*, 2004 ▸). The resonance involving the pyran ring would account for the shortening of the N—N distance through extensive delocalization. The C—N bond lengths [varying between 1.324 (3) and 1.361 (3) Å] are shorter than the normal C—N single bond length (*ca* 1.48 Å), also indicating some degree of delocaliz­ation in both compounds. The S1=C11—N2—N1 torsion angles are 177.31 (16) and 174.29 (16)°, respectively, in mol­ecules *A* and *B* of compound (I) and −172.62 (17)° in compound (II). This indicates that the thionyl atom S1 is positioned *trans* to the azo­methane nitro­gen atom N1 in both compounds.

## Supra­molecular features   

**Figure 3:**
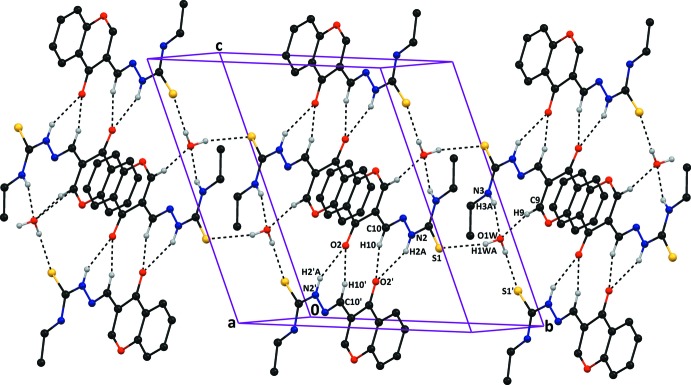
A partial view along the *c* axis of the crystal packing of compound (I), showing the N—H⋯O, C—H⋯O and O*W*—H⋯S hydrogen bonds (dashed lines; see Table 1 ▸), which result in the formation of two 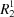
(6) ring motifs and 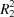
(10), 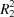
(14), 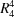
(12) and 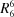
(22) ring motifs. H atoms not involved in hydrogen bonding have been omitted for clarity.

The water mol­ecule of crystallization plays an important role in the hydrogen-bonding patterns of the three-dimensional network in compound (I). In the crystal packing of compound (I), bifurcated N—H⋯O and C—H⋯O hydrogen bonds involving carbonyl oxygens O2 and O2′ in adjacent mol­ecules, inter­connect them to form *A*–*B* dimers with two 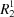
(6) ring motifs, and 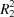
(10) and 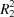
(14) ring motifs (Table 1 ▸ and Fig. 3 ▸). Similar bifurcated hydrogen bonds between mol­ecule *A* and the water O atom form an 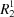
(10) ring motif. In addition to these, the water mol­ecule forms tetra­furcated hydrogen bonds which alternately generate 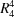
(12) and 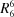
(22) graph-set ring motifs. The supra­molecular aggregation in the crystal of compound (I) is completed by the presence of slipped parallel π–π inter­actions, forming columns along the *c*-axis direction. The most significant inter­actions are *Cg*1⋯*Cg*1^i^ = 3.5648 (14) Å [inter-planar distance = 3.3154 (10) Å, slippage = 1.310 Å, where *Cg*1 is the centroid of the O1/C1/C6–C9 ring; symmetry code: (i) = −*x* + 1, −*y* + 1, −*z* + 1] and *Cg*5⋯*Cg*5^ii^ = 3.6825 (15) Å [inter-planar distance = 3.5441 (11) Å, slippage = 0.999 Å, where *Cg*5 is the centroid of the C1′–C6′ ring; symmetry code: (ii) = −*x* + 2, −*y* + 1, −*z*].

**Figure 4:**
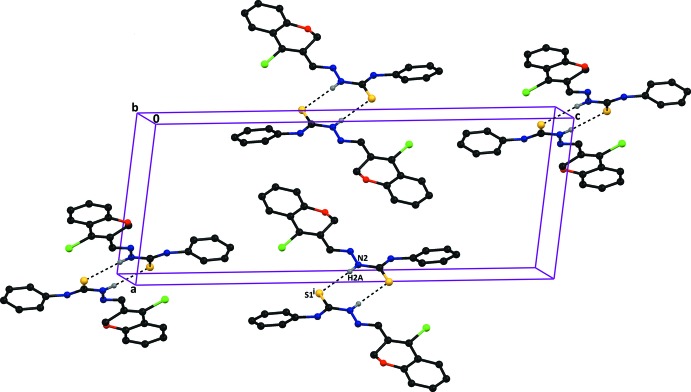
A partial view along the *b* axis of the crystal packing of compound (II), showing the N—H⋯S hydrogen bonds (dashed lines; see Table 2 ▸), which result in the formation of inversion dimers with an 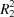
(8) ring motif. H atoms not involved in hydrogen bonding have been omitted for clarity.

In the crystal of compound (II), mol­ecules are linked by pairs of N—H⋯S hydrogen bonds, forming inversion dimers with an 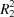
(8) ring motif (Table 2 ▸ and Fig. 4 ▸). The dimers are linked by C—H⋯π inter­actions (Table 2 ▸), forming ribbons lying parallel to plane (210).

## Database survey   

A search of the Cambridge Structural Database (Version 5.36; last update Nov. 2014; Groom & Allen, 2014 ▸) for similar structures gave 3 hits, one of which is a copper(II) complex, di­bromo-(2-{[6-methyl-4-(oxo)-4*H*-chromen-3-yl]methyl­ene}- *N*-phenyl­hydrazinecarbo­thio­amide)­copper (Ilies *et al.*, 2014 ▸). The other two include, *N*-methyl-2-[(4-oxo-4*H*-chromen-3-yl)methyl­ene] hydrazinecarbo­thio­amide (III) (Vimala *et al.*, 2014 ▸), which is the *N*-methyl derivative of compound (I), and (*E*)-2-[(4-chloro-2*H*-chromen-3-yl)methyl­ene]-*N*-cyclo­hexylhydrazine carbo­thio­amide (IV) (Gangadharan *et al.*, 2014 ▸), which is the *N*-cyclo­hexane derivative of compound (II). The bond distances and angles in compounds (I) and (III) are very similar, as are those in compounds (II) and (IV).

## Synthesis and crystallization   

**Compound (I)**: 1.19 g (0.01 mol) of *N*-ethyl­hydrazinecarbo­thio­amide was dissolved in 20 ml of hot ethanol and to this was added 1.74 g (0.01 mol) of 4-oxo-4*H*-chromene-3-carbaldehyde in 10 cm^3^ of ethanol over a period of 10 min with continuous stirring. The reaction mixture was refluxed for 2 h and allowed to cool whereby a shiny white compound began to separate; this was filtered and washed thoroughly with ethanol and then dried *in vacuo*. The compound was recrystallized from hot ethanol (yield: 96%), giving colourless block-like crystals.

**Compound (II)**: 1.67 g (0.01 mol) of 4(*N*)-phenyl­thio­semicarbazide was dissolved in 20 ml of hot ethanol and to this was added 1.94 g (0.01 mol) of 4-chloro-2*H*-chromene-3-carbaldehyde in 10 ml of ethanol over a period of 10 min with continuous stirring. The reaction mixture was refluxed for 2 h and allowed to cool whereby a shiny yellow compound began to separate. It was filtered and washed thoroughly with ethanol and then dried *in vacuo*. The compound was recrystallized from hot ethanol (yield: 89%), giving colourless block-like crystals.

## Refinement   

Crystal data, data collection and structure refinement details for compounds (I) and (II) are summarized in Table 3 ▸. For compound (I), the positions of the water H atoms were located from difference electron density maps and freely refined. In compounds (I) and (II), the NH H atoms were included in calculated positions and treated as riding atoms: N—H = 0.86 Å with *U*_iso_(H) = 1.2*U*_eq_(N). The C-bound H atoms in both mol­ecules were included in calculated positions and treated as riding atoms: C—H = 0.93–0.97 Å with *U*_iso_(H) = 1.5*U*_eq_(C) for methyl H atoms and = 1.2*U*_eq_(C) for other H atoms.

## Supplementary Material

Crystal structure: contains datablock(s) I, II, global. DOI: 10.1107/S2056989015003369/su5078sup1.cif

Structure factors: contains datablock(s) I. DOI: 10.1107/S2056989015003369/su5078Isup2.hkl

Structure factors: contains datablock(s) II. DOI: 10.1107/S2056989015003369/su5078IIsup3.hkl

Supporting information file. DOI: 10.1107/S2056989015003369/su5078Isup4.cml

Supporting information file. DOI: 10.1107/S2056989015003369/su5078IIsup5.cml

CCDC references: 1016440, 1049914

Additional supporting information:  crystallographic information; 3D view; checkCIF report

## Figures and Tables

**Table 1:** Hydrogen-bond geometry (Å, °) for (I)

*D*—H⋯*A*	*D*—H	H⋯*A*	*D*⋯*A*	*D*—H⋯*A*
N2′—H2′*A*⋯O2^i^	0.86	2.09	2.900 (3)	158
N2—H2*A*⋯O2′^i^	0.86	2.14	2.938 (2)	155
N3—H3*A*⋯O1*W*	0.86	2.31	3.131 (3)	161
O1*W*—H1*WB*⋯S1′	0.85 (3)	2.47 (3)	3.322 (2)	178 (4)
O1*W*—H1*WA*⋯S1^ii^	0.87 (2)	2.52 (2)	3.370 (3)	167 (4)
C9—H9⋯O1*W*	0.93	2.30	3.213 (4)	169
C10—H10⋯O2′^i^	0.93	2.51	3.297 (3)	143
C10′—H10′⋯O2^i^	0.93	2.52	3.302 (3)	142

Symmetry codes: (i) 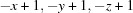; (ii) 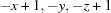.

**Table 2:** Hydrogen-bond geometry (Å, °) for (II) *Cg*1 is the centroid of the C12–C17 phenyl ring.

*D*—H⋯*A*	*D*—H	H⋯*A*	*D*⋯*A*	*D*—H⋯*A*
N2—H2*A*⋯S1^i^	0.86	2.61	3.456 (2)	167
C2—H2⋯*Cg*1^ii^	0.93	2.86	3.697 (3)	151

Symmetry codes: (i) 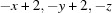; (ii) 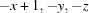.

**Table 3:** Experimental details

	(I)	(II)
Crystal data		
Chemical formula	C_13_H_13_N_3_O_2_S·0.5H_2_O	C_17_H_14_ClN_3_OS
*M*_r_	284.33	343.82
Crystal system, space group	Triclinic, *P* 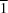	Monoclinic, *P*2_1_/*c*
Temperature (K)	296	296
*a*, *b*, *c* (Å)	8.2858 (2), 12.5422 (4), 14.3520 (5)	10.3176 (3), 5.7589 (2), 27.0364 (7)
α, β, γ (°)	114.379 (2), 95.751 (3), 94.200 (2)	90, 96.564 (2), 90
*V* (Å^3^)	1340.81 (7)	1595.92 (8)
*Z*	4	4
Radiation type	Mo *K*α	Mo *K*α
μ (mm^−1^)	0.25	0.38
Crystal size (mm)	0.35 × 0.30 × 0.25	0.30 × 0.25 × 0.20

Data collection		
Diffractometer	Bruker Kappa APEXII CCD	Bruker Kappa APEXII CCD
Absorption correction	Multi-scan (*SADABS*; Bruker, 2008 ▸)	Multi-scan (*SADABS*; Bruker, 2008 ▸)
*T*_min_, *T*_max_	0.917, 0.940	0.893, 0.927
No. of measured, independent and observed [*I* > 2σ(*I*)] reflections	19142, 5579, 2764	14667, 3902, 2089
*R*_int_	0.046	0.050
(sin θ/λ)_max_ (Å^−1^)	0.631	0.668

Refinement		
*R*[*F* ^2^ > 2σ(*F* ^2^)], *wR*(*F* ^2^), *S*	0.048, 0.137, 0.94	0.047, 0.124, 0.99
No. of reflections	5579	3902
No. of parameters	362	208
No. of restraints	2	0
H-atom treatment	H atoms treated by a mixture of independent and constrained refinement	H-atom parameters constrained
Δρ_max_, Δρ_min_ (e Å^−3^)	0.26, −0.24	0.23, −0.20

Computer programs: *APEX2* and *SAINT* (Bruker, 2008 ▸), *SHELXS97* and *SHELXL97* (Sheldrick, 2008 ▸), *ORTEP-3 for Windows* (Farrugia, 2012 ▸), *Mercury* (Macrae *et al.*, 2008 ▸) and *PLATON* Spek, 2009 ▸).

## supplementary crystallographic information

### (I) (E)-N-Ethyl-2-[(4-oxo-4H-chromen-3-yl)methylidene]hydrazinecarbothioamide hemihydrate Crystal data

**Table:**

C_13_H_13_N_3_O_2_S·0.5H_2_O	*Z* = 4
*M**_r_* = 284.33	*F*(000) = 596
Triclinic, *P*1	*D*_x_ = 1.409 Mg m^−^^3^
Hall symbol: -P 1	Mo *K*α radiation, λ = 0.71073 Å
*a* = 8.2858 (2) Å	Cell parameters from 5579 reflections
*b* = 12.5422 (4) Å	θ = 1.6–26.6°
*c* = 14.3520 (5) Å	µ = 0.25 mm^−^^1^
α = 114.379 (2)°	*T* = 296 K
β = 95.751 (3)°	Block, colourless
γ = 94.200 (2)°	0.35 × 0.30 × 0.25 mm
*V* = 1340.81 (7) Å^3^	

### (I) (E)-N-Ethyl-2-[(4-oxo-4H-chromen-3-yl)methylidene]hydrazinecarbothioamide hemihydrate Data collection

**Table:**

Bruker Kappa APEXII CCD diffractometer	5579 independent reflections
Radiation source: fine-focus sealed tube	2764 reflections with *I* > 2σ(*I*)
Graphite monochromator	*R*_int_ = 0.046
ω and φ scans	θ_max_ = 26.6°, θ_min_ = 1.6°
Absorption correction: multi-scan (*SADABS*; Bruker, 2008)	*h* = −10→10
*T*_min_ = 0.917, *T*_max_ = 0.940	*k* = −14→15
19142 measured reflections	*l* = −18→18

### (I) (E)-N-Ethyl-2-[(4-oxo-4H-chromen-3-yl)methylidene]hydrazinecarbothioamide hemihydrate Refinement

**Table:**

Refinement on *F*^2^	Primary atom site location: structure-invariant direct methods
Least-squares matrix: full	Secondary atom site location: difference Fourier map
*R*[*F*^2^ > 2σ(*F*^2^)] = 0.048	Hydrogen site location: inferred from neighbouring sites
*wR*(*F*^2^) = 0.137	H atoms treated by a mixture of independent and constrained refinement
*S* = 0.94	*w* = 1/[σ^2^(*F*_o_^2^) + (0.0658*P*)^2^] where *P* = (*F*_o_^2^ + 2*F*_c_^2^)/3
5579 reflections	(Δ/σ)_max_ < 0.001
362 parameters	Δρ_max_ = 0.26 e Å^−^^3^
2 restraints	Δρ_min_ = −0.24 e Å^−^^3^

### (I) (E)-N-Ethyl-2-[(4-oxo-4H-chromen-3-yl)methylidene]hydrazinecarbothioamide hemihydrate Special details

**Table:**

Geometry. All e.s.d.'s (except the e.s.d. in the dihedral angle between two l.s. planes) are estimated using the full covariance matrix. The cell e.s.d.'s are taken into account individually in the estimation of e.s.d.'s in distances, angles and torsion angles; correlations between e.s.d.'s in cell parameters are only used when they are defined by crystal symmetry. An approximate (isotropic) treatment of cell e.s.d.'s is used for estimating e.s.d.'s involving l.s. planes.
Refinement. Refinement of *F*^2^ against ALL reflections. The weighted *R*-factor *wR* and goodness of fit *S* are based on *F*^2^, conventional *R*-factors *R* are based on *F*, with *F* set to zero for negative *F*^2^. The threshold expression of *F*^2^ > σ(*F*^2^) is used only for calculating *R*-factors(gt) *etc*. and is not relevant to the choice of reflections for refinement. *R*-factors based on *F*^2^ are statistically about twice as large as those based on *F*, and *R*- factors based on ALL data will be even larger.

### (I) (E)-N-Ethyl-2-[(4-oxo-4H-chromen-3-yl)methylidene]hydrazinecarbothioamide hemihydrate Fractional atomic coordinates and isotropic or equivalent isotropic displacement parameters (Å^2^)

**Table:**

	*x*	*y*	*z*	*U*_iso_*/*U*_eq_	
C1'	0.7753 (3)	0.4656 (2)	−0.08664 (19)	0.0444 (6)	
C1	0.8004 (3)	0.5298 (2)	0.46364 (19)	0.0425 (6)	
C2'	0.8576 (3)	0.5059 (2)	−0.1473 (2)	0.0557 (7)	
H2'	0.8603	0.4568	−0.2163	0.067*	
C2	0.8858 (3)	0.5665 (2)	0.4025 (2)	0.0557 (7)	
H2	0.8976	0.5138	0.3360	0.067*	
C3	0.9530 (3)	0.6821 (2)	0.4413 (2)	0.0581 (7)	
H3	1.0107	0.7083	0.4008	0.070*	
C3'	0.9351 (3)	0.6189 (2)	−0.1045 (2)	0.0593 (8)	
H3'	0.9903	0.6469	−0.1448	0.071*	
C4'	0.9321 (3)	0.6925 (2)	−0.0009 (2)	0.0572 (7)	
H4'	0.9859	0.7690	0.0278	0.069*	
C4	0.9357 (3)	0.7602 (2)	0.5402 (2)	0.0568 (7)	
H4	0.9835	0.8382	0.5664	0.068*	
C5	0.8487 (3)	0.7233 (2)	0.6000 (2)	0.0487 (6)	
H5	0.8357	0.7768	0.6660	0.058*	
C5'	0.8498 (3)	0.6520 (2)	0.0583 (2)	0.0503 (7)	
H5'	0.8475	0.7017	0.1273	0.060*	
C6	0.7792 (3)	0.6059 (2)	0.56270 (18)	0.0404 (6)	
C6'	0.7691 (3)	0.5370 (2)	0.01658 (19)	0.0408 (6)	
C7	0.6827 (3)	0.5632 (2)	0.62307 (19)	0.0420 (6)	
C7'	0.6784 (3)	0.4911 (2)	0.07702 (19)	0.0429 (6)	
C8'	0.6037 (3)	0.3694 (2)	0.02285 (18)	0.0409 (6)	
C8	0.6228 (3)	0.43815 (19)	0.57379 (18)	0.0394 (6)	
C9	0.6516 (3)	0.3722 (2)	0.47741 (19)	0.0488 (7)	
H9	0.6102	0.2926	0.4477	0.059*	
C9'	0.6189 (3)	0.3082 (2)	−0.0777 (2)	0.0535 (7)	
H9'	0.5693	0.2305	−0.1106	0.064*	
C10	0.5298 (3)	0.3872 (2)	0.62971 (18)	0.0430 (6)	
H10	0.5125	0.4350	0.6965	0.052*	
C10'	0.5137 (3)	0.3172 (2)	0.07880 (19)	0.0456 (6)	
H10'	0.5090	0.3620	0.1485	0.055*	
C11	0.3128 (3)	0.1280 (2)	0.61075 (18)	0.0412 (6)	
C11'	0.2875 (3)	0.0607 (2)	0.05910 (19)	0.0420 (6)	
C12	0.2363 (3)	−0.0610 (2)	0.45867 (19)	0.0538 (7)	
H12A	0.1249	−0.0630	0.4739	0.065*	
H12B	0.2923	−0.1109	0.4842	0.065*	
C12'	0.1908 (3)	−0.1203 (2)	−0.09642 (19)	0.0524 (7)	
H12C	0.0750	−0.1186	−0.0915	0.063*	
H12D	0.2316	−0.1696	−0.0645	0.063*	
C13'	0.2150 (4)	−0.1715 (2)	−0.2081 (2)	0.0732 (9)	
H13D	0.1711	−0.1241	−0.2402	0.110*	
H13E	0.1596	−0.2505	−0.2426	0.110*	
H13F	0.3296	−0.1725	−0.2128	0.110*	
C13	0.2336 (3)	−0.1086 (2)	0.3439 (2)	0.0631 (8)	
H13A	0.1773	−0.0598	0.3182	0.095*	
H13B	0.1782	−0.1877	0.3115	0.095*	
H13C	0.3437	−0.1086	0.3285	0.095*	
N1	0.4719 (2)	0.27888 (16)	0.58919 (15)	0.0431 (5)	
N1'	0.4412 (2)	0.21203 (17)	0.03521 (15)	0.0447 (5)	
N2'	0.3664 (2)	0.17184 (16)	0.09748 (16)	0.0499 (5)	
H2'A	0.3695	0.2178	0.1618	0.060*	
N2	0.3884 (2)	0.24022 (16)	0.64915 (15)	0.0454 (5)	
H2A	0.3837	0.2878	0.7121	0.055*	
N3'	0.2766 (2)	−0.00132 (17)	−0.04207 (16)	0.0511 (6)	
H3'A	0.3224	0.0303	−0.0773	0.061*	
N3	0.3184 (2)	0.06031 (16)	0.51214 (15)	0.0475 (5)	
H3A	0.3728	0.0889	0.4779	0.057*	
O1	0.7355 (2)	0.41240 (14)	0.42089 (13)	0.0534 (5)	
O1'	0.6999 (2)	0.35106 (14)	−0.13347 (13)	0.0575 (5)	
O2	0.6529 (2)	0.62856 (14)	0.70913 (13)	0.0603 (5)	
O2'	0.6649 (2)	0.55142 (14)	0.16840 (14)	0.0644 (5)	
O1W	0.4956 (3)	0.10193 (19)	0.34506 (17)	0.0689 (6)	
S1'	0.21192 (9)	0.00898 (6)	0.13833 (5)	0.0591 (2)	
S1	0.21930 (9)	0.08415 (6)	0.68995 (5)	0.0587 (2)	
H1WA	0.569 (4)	0.054 (3)	0.326 (4)	0.18 (2)*	
H1WB	0.422 (4)	0.080 (4)	0.293 (2)	0.16 (2)*	

### (I) (E)-N-Ethyl-2-[(4-oxo-4H-chromen-3-yl)methylidene]hydrazinecarbothioamide hemihydrate Atomic displacement parameters (Å^2^)

**Table:**

	*U*^11^	*U*^22^	*U*^33^	*U*^12^	*U*^13^	*U*^23^
C1'	0.0496 (15)	0.0441 (15)	0.0419 (16)	0.0038 (12)	0.0133 (13)	0.0194 (13)
C1	0.0458 (15)	0.0384 (14)	0.0450 (16)	0.0025 (12)	0.0109 (13)	0.0186 (13)
C2'	0.0640 (17)	0.0635 (18)	0.0423 (16)	0.0010 (15)	0.0162 (14)	0.0244 (15)
C2	0.0649 (18)	0.0576 (18)	0.0497 (17)	0.0082 (15)	0.0259 (15)	0.0238 (15)
C3	0.0645 (18)	0.0609 (18)	0.0604 (19)	0.0022 (15)	0.0229 (15)	0.0348 (16)
C3'	0.0595 (18)	0.0645 (19)	0.063 (2)	−0.0021 (15)	0.0179 (15)	0.0363 (17)
C4'	0.0574 (17)	0.0525 (17)	0.064 (2)	−0.0023 (14)	0.0111 (15)	0.0284 (16)
C4	0.0595 (17)	0.0492 (16)	0.064 (2)	−0.0055 (14)	0.0125 (15)	0.0278 (16)
C5	0.0547 (16)	0.0430 (15)	0.0467 (16)	−0.0020 (13)	0.0092 (13)	0.0181 (13)
C5'	0.0566 (16)	0.0463 (15)	0.0475 (16)	0.0018 (13)	0.0113 (13)	0.0193 (13)
C6	0.0421 (14)	0.0411 (14)	0.0404 (15)	0.0030 (11)	0.0084 (12)	0.0194 (12)
C6'	0.0441 (14)	0.0392 (14)	0.0422 (15)	0.0051 (11)	0.0096 (12)	0.0196 (12)
C7	0.0483 (15)	0.0415 (14)	0.0366 (15)	0.0032 (12)	0.0064 (12)	0.0172 (13)
C7'	0.0481 (15)	0.0405 (14)	0.0411 (16)	0.0068 (12)	0.0129 (13)	0.0167 (13)
C8'	0.0460 (14)	0.0385 (14)	0.0389 (15)	0.0040 (11)	0.0113 (12)	0.0162 (12)
C8	0.0462 (14)	0.0349 (13)	0.0372 (15)	0.0031 (11)	0.0099 (12)	0.0150 (12)
C9	0.0612 (17)	0.0384 (14)	0.0463 (16)	−0.0024 (13)	0.0167 (14)	0.0170 (13)
C9'	0.0697 (18)	0.0418 (15)	0.0472 (17)	−0.0033 (13)	0.0186 (14)	0.0163 (14)
C10	0.0531 (15)	0.0389 (14)	0.0365 (14)	0.0013 (12)	0.0115 (12)	0.0151 (12)
C10'	0.0574 (16)	0.0398 (14)	0.0401 (15)	0.0045 (13)	0.0167 (13)	0.0156 (13)
C11	0.0483 (15)	0.0353 (14)	0.0417 (15)	0.0055 (12)	0.0119 (12)	0.0169 (12)
C11'	0.0468 (15)	0.0358 (14)	0.0432 (16)	0.0027 (12)	0.0100 (12)	0.0160 (12)
C12	0.0696 (18)	0.0405 (14)	0.0489 (17)	−0.0031 (13)	0.0164 (14)	0.0164 (13)
C12'	0.0598 (17)	0.0452 (15)	0.0488 (17)	−0.0027 (13)	0.0110 (14)	0.0174 (13)
C13'	0.094 (2)	0.0668 (19)	0.0442 (18)	−0.0098 (17)	0.0102 (17)	0.0126 (15)
C13	0.0741 (19)	0.0524 (17)	0.0506 (18)	−0.0046 (15)	0.0149 (15)	0.0105 (14)
N1	0.0539 (13)	0.0379 (12)	0.0395 (12)	−0.0002 (10)	0.0126 (10)	0.0182 (10)
N1'	0.0547 (13)	0.0400 (12)	0.0417 (12)	0.0007 (10)	0.0154 (10)	0.0184 (10)
N2'	0.0680 (14)	0.0413 (12)	0.0389 (12)	−0.0031 (11)	0.0175 (11)	0.0150 (10)
N2	0.0614 (13)	0.0371 (11)	0.0368 (12)	−0.0003 (10)	0.0166 (10)	0.0136 (10)
N3'	0.0663 (14)	0.0437 (12)	0.0412 (13)	−0.0063 (11)	0.0148 (11)	0.0164 (11)
N3	0.0602 (13)	0.0403 (12)	0.0422 (13)	−0.0013 (10)	0.0180 (11)	0.0166 (10)
O1	0.0716 (12)	0.0426 (10)	0.0428 (11)	−0.0008 (9)	0.0246 (9)	0.0125 (9)
O1'	0.0788 (13)	0.0477 (11)	0.0407 (11)	−0.0064 (9)	0.0234 (10)	0.0127 (9)
O2	0.0948 (14)	0.0418 (10)	0.0390 (11)	−0.0025 (10)	0.0261 (10)	0.0101 (9)
O2'	0.0966 (14)	0.0463 (11)	0.0427 (12)	−0.0063 (10)	0.0302 (11)	0.0094 (9)
O1W	0.0823 (16)	0.0636 (14)	0.0546 (14)	−0.0030 (13)	0.0150 (13)	0.0196 (11)
S1'	0.0820 (5)	0.0521 (4)	0.0441 (4)	−0.0073 (4)	0.0147 (4)	0.0227 (4)
S1	0.0800 (5)	0.0487 (4)	0.0507 (4)	−0.0004 (4)	0.0288 (4)	0.0214 (3)

### (I) (E)-N-Ethyl-2-[(4-oxo-4H-chromen-3-yl)methylidene]hydrazinecarbothioamide hemihydrate Geometric parameters (Å, º)

**Table:**

C1'—O1'	1.376 (3)	C9'—O1'	1.338 (3)
C1'—C2'	1.382 (3)	C9'—H9'	0.9300
C1'—C6'	1.388 (3)	C10—N1	1.269 (3)
C1—C2	1.376 (3)	C10—H10	0.9300
C1—O1	1.380 (3)	C10'—N1'	1.273 (3)
C1—C6	1.385 (3)	C10'—H10'	0.9300
C2'—C3'	1.366 (3)	C11—N3	1.324 (3)
C2'—H2'	0.9300	C11—N2	1.356 (3)
C2—C3	1.370 (3)	C11—S1	1.681 (2)
C2—H2	0.9300	C11'—N3'	1.324 (3)
C3—C4	1.382 (4)	C11'—N2'	1.354 (3)
C3—H3	0.9300	C11'—S1'	1.673 (2)
C3'—C4'	1.392 (4)	C12—N3	1.465 (3)
C3'—H3'	0.9300	C12—C13	1.500 (3)
C4'—C5'	1.367 (3)	C12—H12A	0.9700
C4'—H4'	0.9300	C12—H12B	0.9700
C4—C5	1.369 (3)	C12'—N3'	1.454 (3)
C4—H4	0.9300	C12'—C13'	1.501 (3)
C5—C6	1.396 (3)	C12'—H12C	0.9700
C5—H5	0.9300	C12'—H12D	0.9700
C5'—C6'	1.398 (3)	C13'—H13D	0.9600
C5'—H5'	0.9300	C13'—H13E	0.9600
C6—C7	1.461 (3)	C13'—H13F	0.9600
C6'—C7'	1.458 (3)	C13—H13A	0.9600
C7—O2	1.230 (3)	C13—H13B	0.9600
C7—C8	1.452 (3)	C13—H13C	0.9600
C7'—O2'	1.236 (3)	N1—N2	1.367 (2)
C7'—C8'	1.451 (3)	N1'—N2'	1.369 (2)
C8'—C9'	1.350 (3)	N2'—H2'A	0.8600
C8'—C10'	1.453 (3)	N2—H2A	0.8600
C8—C9	1.344 (3)	N3'—H3'A	0.8600
C8—C10	1.457 (3)	N3—H3A	0.8600
C9—O1	1.339 (2)	O1W—H1WA	0.866 (19)
C9—H9	0.9300	O1W—H1WB	0.847 (19)
			
O1'—C1'—C2'	116.8 (2)	N1—C10—C8	121.4 (2)
O1'—C1'—C6'	121.5 (2)	N1—C10—H10	119.3
C2'—C1'—C6'	121.7 (2)	C8—C10—H10	119.3
C2—C1—O1	116.5 (2)	N1'—C10'—C8'	121.9 (2)
C2—C1—C6	122.3 (2)	N1'—C10'—H10'	119.0
O1—C1—C6	121.3 (2)	C8'—C10'—H10'	119.0
C3'—C2'—C1'	119.2 (2)	N3—C11—N2	116.8 (2)
C3'—C2'—H2'	120.4	N3—C11—S1	124.54 (18)
C1'—C2'—H2'	120.4	N2—C11—S1	118.62 (18)
C3—C2—C1	118.8 (2)	N3'—C11'—N2'	116.0 (2)
C3—C2—H2	120.6	N3'—C11'—S1'	123.86 (18)
C1—C2—H2	120.6	N2'—C11'—S1'	120.18 (19)
C2—C3—C4	120.5 (2)	N3—C12—C13	111.8 (2)
C2—C3—H3	119.8	N3—C12—H12A	109.2
C4—C3—H3	119.8	C13—C12—H12A	109.2
C2'—C3'—C4'	120.6 (2)	N3—C12—H12B	109.2
C2'—C3'—H3'	119.7	C13—C12—H12B	109.2
C4'—C3'—H3'	119.7	H12A—C12—H12B	107.9
C5'—C4'—C3'	119.9 (2)	N3'—C12'—C13'	110.4 (2)
C5'—C4'—H4'	120.1	N3'—C12'—H12C	109.6
C3'—C4'—H4'	120.1	C13'—C12'—H12C	109.6
C5—C4—C3	120.3 (2)	N3'—C12'—H12D	109.6
C5—C4—H4	119.8	C13'—C12'—H12D	109.6
C3—C4—H4	119.8	H12C—C12'—H12D	108.1
C4—C5—C6	120.5 (2)	C12'—C13'—H13D	109.5
C4—C5—H5	119.7	C12'—C13'—H13E	109.5
C6—C5—H5	119.7	H13D—C13'—H13E	109.5
C4'—C5'—C6'	120.8 (2)	C12'—C13'—H13F	109.5
C4'—C5'—H5'	119.6	H13D—C13'—H13F	109.5
C6'—C5'—H5'	119.6	H13E—C13'—H13F	109.5
C1—C6—C5	117.6 (2)	C12—C13—H13A	109.5
C1—C6—C7	120.0 (2)	C12—C13—H13B	109.5
C5—C6—C7	122.3 (2)	H13A—C13—H13B	109.5
C1'—C6'—C5'	117.9 (2)	C12—C13—H13C	109.5
C1'—C6'—C7'	119.7 (2)	H13A—C13—H13C	109.5
C5'—C6'—C7'	122.4 (2)	H13B—C13—H13C	109.5
O2—C7—C8	122.2 (2)	C10—N1—N2	116.40 (19)
O2—C7—C6	122.6 (2)	C10'—N1'—N2'	116.1 (2)
C8—C7—C6	115.2 (2)	C11'—N2'—N1'	120.9 (2)
O2'—C7'—C8'	121.7 (2)	C11'—N2'—H2'A	119.6
O2'—C7'—C6'	122.7 (2)	N1'—N2'—H2'A	119.6
C8'—C7'—C6'	115.7 (2)	C11—N2—N1	120.95 (19)
C9'—C8'—C7'	119.3 (2)	C11—N2—H2A	119.5
C9'—C8'—C10'	122.0 (2)	N1—N2—H2A	119.5
C7'—C8'—C10'	118.7 (2)	C11'—N3'—C12'	123.2 (2)
C9—C8—C7	119.7 (2)	C11'—N3'—H3'A	118.4
C9—C8—C10	121.4 (2)	C12'—N3'—H3'A	118.4
C7—C8—C10	119.0 (2)	C11—N3—C12	123.1 (2)
O1—C9—C8	125.0 (2)	C11—N3—H3A	118.5
O1—C9—H9	117.5	C12—N3—H3A	118.5
C8—C9—H9	117.5	C9—O1—C1	118.76 (18)
O1'—C9'—C8'	124.9 (2)	C9'—O1'—C1'	118.85 (19)
O1'—C9'—H9'	117.5	H1WA—O1W—H1WB	106 (4)
C8'—C9'—H9'	117.5		
			
O1'—C1'—C2'—C3'	−179.8 (2)	C6'—C7'—C8'—C10'	179.0 (2)
C6'—C1'—C2'—C3'	0.1 (4)	O2—C7—C8—C9	176.4 (2)
O1—C1—C2—C3	−179.9 (2)	C6—C7—C8—C9	−2.9 (3)
C6—C1—C2—C3	0.5 (4)	O2—C7—C8—C10	−2.5 (4)
C1—C2—C3—C4	0.2 (4)	C6—C7—C8—C10	178.11 (19)
C1'—C2'—C3'—C4'	0.3 (4)	C7—C8—C9—O1	0.9 (4)
C2'—C3'—C4'—C5'	−0.6 (4)	C10—C8—C9—O1	179.8 (2)
C2—C3—C4—C5	−1.1 (4)	C7'—C8'—C9'—O1'	0.3 (4)
C3—C4—C5—C6	1.3 (4)	C10'—C8'—C9'—O1'	180.0 (2)
C3'—C4'—C5'—C6'	0.4 (4)	C9—C8—C10—N1	−0.4 (4)
C2—C1—C6—C5	−0.3 (4)	C7—C8—C10—N1	178.5 (2)
O1—C1—C6—C5	−179.9 (2)	C9'—C8'—C10'—N1'	−1.7 (4)
C2—C1—C6—C7	177.9 (2)	C7'—C8'—C10'—N1'	177.9 (2)
O1—C1—C6—C7	−1.7 (4)	C8—C10—N1—N2	179.22 (18)
C4—C5—C6—C1	−0.6 (4)	C8'—C10'—N1'—N2'	177.44 (19)
C4—C5—C6—C7	−178.8 (2)	N3'—C11'—N2'—N1'	−5.3 (3)
O1'—C1'—C6'—C5'	179.6 (2)	S1'—C11'—N2'—N1'	174.29 (16)
C2'—C1'—C6'—C5'	−0.2 (4)	C10'—N1'—N2'—C11'	−179.2 (2)
O1'—C1'—C6'—C7'	−1.1 (4)	N3—C11—N2—N1	−2.7 (3)
C2'—C1'—C6'—C7'	179.0 (2)	S1—C11—N2—N1	177.31 (16)
C4'—C5'—C6'—C1'	0.0 (4)	C10—N1—N2—C11	175.7 (2)
C4'—C5'—C6'—C7'	−179.3 (2)	N2'—C11'—N3'—C12'	−177.6 (2)
C1—C6—C7—O2	−176.0 (2)	S1'—C11'—N3'—C12'	2.8 (3)
C5—C6—C7—O2	2.1 (4)	C13'—C12'—N3'—C11'	−174.1 (2)
C1—C6—C7—C8	3.3 (3)	N2—C11—N3—C12	−177.1 (2)
C5—C6—C7—C8	−178.5 (2)	S1—C11—N3—C12	2.8 (3)
C1'—C6'—C7'—O2'	−178.0 (2)	C13—C12—N3—C11	168.4 (2)
C5'—C6'—C7'—O2'	1.2 (4)	C8—C9—O1—C1	1.0 (4)
C1'—C6'—C7'—C8'	1.7 (3)	C2—C1—O1—C9	179.8 (2)
C5'—C6'—C7'—C8'	−179.1 (2)	C6—C1—O1—C9	−0.5 (3)
O2'—C7'—C8'—C9'	178.4 (2)	C8'—C9'—O1'—C1'	0.4 (4)
C6'—C7'—C8'—C9'	−1.3 (3)	C2'—C1'—O1'—C9'	179.9 (2)
O2'—C7'—C8'—C10'	−1.3 (3)	C6'—C1'—O1'—C9'	0.1 (3)

### (I) (E)-N-Ethyl-2-[(4-oxo-4H-chromen-3-yl)methylidene]hydrazinecarbothioamide hemihydrate Hydrogen-bond geometry (Å, º)

**Table:**

*D*—H···*A*	*D*—H	H···*A*	*D*···*A*	*D*—H···*A*
N2′—H2′*A*···O2^i^	0.86	2.09	2.900 (3)	158
N2—H2*A*···O2′^i^	0.86	2.14	2.938 (2)	155
N3—H3*A*···O1*W*	0.86	2.31	3.131 (3)	161
O1*W*—H1*WB*···S1′	0.85 (3)	2.47 (3)	3.322 (2)	178 (4)
O1*W*—H1*WA*···S1^ii^	0.87 (2)	2.52 (2)	3.370 (3)	167 (4)
C9—H9···O1*W*	0.93	2.30	3.213 (4)	169
C10—H10···O2′^i^	0.93	2.51	3.297 (3)	143
C10′—H10′···O2^i^	0.93	2.52	3.302 (3)	142

Symmetry codes: (i) −*x*+1, −*y*+1, −*z*+1; (ii) −*x*+1, −*y*, −*z*+1.

### (II) (E)-2-[(4-Chloro-2H-chromen-3-yl)methylidene]-N-phenylhydrazinecarbothioamide Crystal data

**Table:**

C_17_H_14_ClN_3_OS	*F*(000) = 712
*M**_r_* = 343.82	*D*_x_ = 1.431 Mg m^−^^3^
Monoclinic, *P*2_1_/*c*	Mo *K*α radiation, λ = 0.71073 Å
Hall symbol: -P 2ybc	Cell parameters from 3902 reflections
*a* = 10.3176 (3) Å	θ = 1.5–28.4°
*b* = 5.7589 (2) Å	µ = 0.38 mm^−^^1^
*c* = 27.0364 (7) Å	*T* = 296 K
β = 96.564 (2)°	Block, colourless
*V* = 1595.92 (8) Å^3^	0.30 × 0.25 × 0.20 mm
*Z* = 4	

### (II) (E)-2-[(4-Chloro-2H-chromen-3-yl)methylidene]-N-phenylhydrazinecarbothioamide Data collection

**Table:**

Bruker Kappa APEXII CCD diffractometer	3902 independent reflections
Radiation source: fine-focus sealed tube	2089 reflections with *I* > 2σ(*I*)
Graphite monochromator	*R*_int_ = 0.050
ω and φ scans	θ_max_ = 28.4°, θ_min_ = 1.5°
Absorption correction: multi-scan (*SADABS*; Bruker, 2008)	*h* = −13→13
*T*_min_ = 0.893, *T*_max_ = 0.927	*k* = −7→7
14667 measured reflections	*l* = −35→35

### (II) (E)-2-[(4-Chloro-2H-chromen-3-yl)methylidene]-N-phenylhydrazinecarbothioamide Refinement

**Table:**

Refinement on *F*^2^	Primary atom site location: structure-invariant direct methods
Least-squares matrix: full	Secondary atom site location: difference Fourier map
*R*[*F*^2^ > 2σ(*F*^2^)] = 0.047	Hydrogen site location: inferred from neighbouring sites
*wR*(*F*^2^) = 0.124	H-atom parameters constrained
*S* = 0.99	*w* = 1/[σ^2^(*F*_o_^2^) + (0.053*P*)^2^ + 0.0466*P*] where *P* = (*F*_o_^2^ + 2*F*_c_^2^)/3
3902 reflections	(Δ/σ)_max_ < 0.001
208 parameters	Δρ_max_ = 0.23 e Å^−^^3^
0 restraints	Δρ_min_ = −0.20 e Å^−^^3^

### (II) (E)-2-[(4-Chloro-2H-chromen-3-yl)methylidene]-N-phenylhydrazinecarbothioamide Special details

**Table:**

Geometry. All e.s.d.'s (except the e.s.d. in the dihedral angle between two l.s. planes) are estimated using the full covariance matrix. The cell e.s.d.'s are taken into account individually in the estimation of e.s.d.'s in distances, angles and torsion angles; correlations between e.s.d.'s in cell parameters are only used when they are defined by crystal symmetry. An approximate (isotropic) treatment of cell e.s.d.'s is used for estimating e.s.d.'s involving l.s. planes.
Refinement. Refinement of *F*^2^ against ALL reflections. The weighted *R*-factor *wR* and goodness of fit *S* are based on *F*^2^, conventional *R*-factors *R* are based on *F*, with *F* set to zero for negative *F*^2^. The threshold expression of *F*^2^ > σ(*F*^2^) is used only for calculating *R*-factors(gt) *etc*. and is not relevant to the choice of reflections for refinement. *R*-factors based on *F*^2^ are statistically about twice as large as those based on *F*, and *R*- factors based on ALL data will be even larger.

### (II) (E)-2-[(4-Chloro-2H-chromen-3-yl)methylidene]-N-phenylhydrazinecarbothioamide Fractional atomic coordinates and isotropic or equivalent isotropic displacement parameters (Å^2^)

**Table:**

	*x*	*y*	*z*	*U*_iso_*/*U*_eq_	
C1	0.4918 (3)	−0.2996 (5)	−0.10347 (12)	0.0665 (8)	
H1	0.4591	−0.3879	−0.0789	0.080*	
C2	0.4637 (3)	−0.3582 (5)	−0.15313 (13)	0.0749 (9)	
H2	0.4120	−0.4872	−0.1621	0.090*	
C3	0.5116 (3)	−0.2267 (6)	−0.18911 (12)	0.0759 (9)	
H3	0.4921	−0.2669	−0.2224	0.091*	
C4	0.5882 (3)	−0.0365 (5)	−0.17654 (10)	0.0622 (7)	
H4	0.6193	0.0518	−0.2015	0.075*	
C5	0.6198 (2)	0.0260 (4)	−0.12710 (8)	0.0457 (6)	
C6	0.5690 (2)	−0.1087 (4)	−0.09079 (10)	0.0531 (6)	
C7	0.6397 (3)	0.1634 (4)	−0.02714 (9)	0.0563 (7)	
H7A	0.6881	0.1612	0.0058	0.068*	
H7B	0.5608	0.2529	−0.0251	0.068*	
C8	0.7205 (2)	0.2839 (4)	−0.06223 (8)	0.0456 (6)	
C9	0.7056 (2)	0.2157 (4)	−0.11002 (8)	0.0446 (6)	
C10	0.7994 (2)	0.4764 (4)	−0.04404 (9)	0.0490 (6)	
H10	0.8449	0.5619	−0.0656	0.059*	
C11	0.8930 (2)	0.7961 (4)	0.06460 (8)	0.0458 (6)	
C12	0.8439 (2)	0.6739 (4)	0.14825 (8)	0.0430 (6)	
C13	0.8894 (2)	0.4948 (4)	0.17950 (9)	0.0497 (6)	
H13	0.9269	0.3642	0.1667	0.060*	
C14	0.8788 (2)	0.5110 (5)	0.22994 (9)	0.0538 (7)	
H14	0.9088	0.3902	0.2510	0.065*	
C15	0.8245 (2)	0.7033 (5)	0.24904 (9)	0.0563 (7)	
H15	0.8189	0.7146	0.2831	0.068*	
C16	0.7784 (2)	0.8787 (5)	0.21777 (9)	0.0580 (7)	
H16	0.7407	1.0086	0.2307	0.070*	
C17	0.7870 (2)	0.8659 (4)	0.16725 (9)	0.0520 (6)	
H17	0.7547	0.9856	0.1463	0.062*	
N1	0.80705 (19)	0.5309 (3)	0.00252 (7)	0.0496 (5)	
N2	0.87785 (19)	0.7257 (4)	0.01625 (7)	0.0533 (5)	
H2A	0.9130	0.8041	−0.0058	0.064*	
N3	0.84862 (19)	0.6451 (3)	0.09615 (7)	0.0520 (5)	
H3A	0.8196	0.5154	0.0836	0.062*	
O1	0.60439 (18)	−0.0669 (3)	−0.04098 (6)	0.0662 (5)	
Cl1	0.79000 (7)	0.35034 (13)	−0.15374 (2)	0.0649 (2)	
S1	0.96413 (7)	1.05093 (12)	0.07927 (2)	0.0565 (2)	

### (II) (E)-2-[(4-Chloro-2H-chromen-3-yl)methylidene]-N-phenylhydrazinecarbothioamide Atomic displacement parameters (Å^2^)

**Table:**

	*U*^11^	*U*^22^	*U*^33^	*U*^12^	*U*^13^	*U*^23^
C1	0.0604 (16)	0.0548 (19)	0.086 (2)	−0.0045 (14)	0.0171 (15)	−0.0053 (17)
C2	0.0523 (16)	0.070 (2)	0.101 (3)	−0.0045 (15)	0.0005 (16)	−0.030 (2)
C3	0.0651 (18)	0.092 (3)	0.068 (2)	0.0024 (18)	−0.0059 (15)	−0.0280 (19)
C4	0.0606 (16)	0.074 (2)	0.0509 (16)	0.0048 (15)	0.0005 (13)	−0.0125 (15)
C5	0.0467 (13)	0.0493 (15)	0.0410 (13)	0.0075 (11)	0.0046 (10)	−0.0025 (12)
C6	0.0534 (14)	0.0502 (17)	0.0564 (16)	0.0037 (12)	0.0089 (12)	−0.0055 (13)
C7	0.0773 (17)	0.0502 (17)	0.0437 (14)	−0.0077 (14)	0.0160 (12)	0.0006 (13)
C8	0.0527 (13)	0.0474 (15)	0.0375 (13)	0.0009 (12)	0.0093 (10)	0.0022 (12)
C9	0.0500 (13)	0.0473 (15)	0.0374 (12)	0.0027 (11)	0.0096 (10)	0.0037 (11)
C10	0.0584 (15)	0.0506 (16)	0.0387 (13)	−0.0022 (12)	0.0088 (11)	0.0028 (12)
C11	0.0528 (14)	0.0441 (15)	0.0416 (13)	0.0011 (11)	0.0103 (11)	0.0004 (12)
C12	0.0496 (13)	0.0409 (15)	0.0397 (13)	−0.0090 (11)	0.0099 (10)	−0.0015 (11)
C13	0.0582 (15)	0.0412 (15)	0.0522 (15)	0.0008 (12)	0.0167 (12)	−0.0038 (12)
C14	0.0631 (16)	0.0518 (17)	0.0479 (15)	−0.0023 (13)	0.0123 (12)	0.0063 (13)
C15	0.0646 (16)	0.0636 (19)	0.0428 (14)	−0.0140 (14)	0.0156 (12)	−0.0079 (14)
C16	0.0673 (16)	0.0512 (17)	0.0589 (17)	−0.0020 (13)	0.0211 (13)	−0.0143 (14)
C17	0.0623 (16)	0.0413 (15)	0.0539 (15)	0.0029 (12)	0.0128 (12)	0.0021 (13)
N1	0.0624 (13)	0.0435 (13)	0.0433 (12)	−0.0043 (10)	0.0083 (9)	−0.0006 (10)
N2	0.0713 (13)	0.0527 (14)	0.0368 (11)	−0.0131 (11)	0.0101 (9)	0.0021 (10)
N3	0.0789 (14)	0.0406 (12)	0.0386 (11)	−0.0136 (11)	0.0148 (10)	−0.0063 (10)
O1	0.0928 (13)	0.0550 (13)	0.0525 (11)	−0.0160 (10)	0.0162 (10)	0.0034 (10)
Cl1	0.0802 (5)	0.0762 (5)	0.0411 (4)	−0.0098 (4)	0.0189 (3)	0.0059 (3)
S1	0.0796 (5)	0.0433 (4)	0.0488 (4)	−0.0101 (3)	0.0162 (3)	−0.0023 (3)

### (II) (E)-2-[(4-Chloro-2H-chromen-3-yl)methylidene]-N-phenylhydrazinecarbothioamide Geometric parameters (Å, º)

**Table:**

C1—C6	1.378 (3)	C10—H10	0.9300
C1—C2	1.383 (4)	C11—N3	1.335 (3)
C1—H1	0.9300	C11—N2	1.361 (3)
C2—C3	1.369 (4)	C11—S1	1.668 (2)
C2—H2	0.9300	C12—C17	1.378 (3)
C3—C4	1.371 (4)	C12—C13	1.381 (3)
C3—H3	0.9300	C12—N3	1.425 (3)
C4—C5	1.387 (3)	C13—C14	1.384 (3)
C4—H4	0.9300	C13—H13	0.9300
C5—C6	1.399 (3)	C14—C15	1.369 (3)
C5—C9	1.448 (3)	C14—H14	0.9300
C6—O1	1.375 (3)	C15—C16	1.367 (3)
C7—O1	1.414 (3)	C15—H15	0.9300
C7—C8	1.503 (3)	C16—C17	1.381 (3)
C7—H7A	0.9700	C16—H16	0.9300
C7—H7B	0.9700	C17—H17	0.9300
C8—C9	1.343 (3)	N1—N2	1.367 (3)
C8—C10	1.429 (3)	N2—H2A	0.8600
C9—Cl1	1.730 (2)	N3—H3A	0.8600
C10—N1	1.291 (3)		
			
C6—C1—C2	119.2 (3)	N1—C10—H10	120.2
C6—C1—H1	120.4	C8—C10—H10	120.2
C2—C1—H1	120.4	N3—C11—N2	114.2 (2)
C3—C2—C1	120.1 (3)	N3—C11—S1	126.51 (18)
C3—C2—H2	119.9	N2—C11—S1	119.31 (18)
C1—C2—H2	119.9	C17—C12—C13	120.0 (2)
C2—C3—C4	120.7 (3)	C17—C12—N3	121.7 (2)
C2—C3—H3	119.6	C13—C12—N3	118.1 (2)
C4—C3—H3	119.6	C12—C13—C14	119.5 (2)
C3—C4—C5	120.8 (3)	C12—C13—H13	120.2
C3—C4—H4	119.6	C14—C13—H13	120.2
C5—C4—H4	119.6	C15—C14—C13	120.6 (2)
C4—C5—C6	117.8 (2)	C15—C14—H14	119.7
C4—C5—C9	124.8 (2)	C13—C14—H14	119.7
C6—C5—C9	117.3 (2)	C16—C15—C14	119.5 (2)
O1—C6—C1	117.7 (3)	C16—C15—H15	120.2
O1—C6—C5	120.8 (2)	C14—C15—H15	120.2
C1—C6—C5	121.3 (3)	C15—C16—C17	121.0 (2)
O1—C7—C8	114.2 (2)	C15—C16—H16	119.5
O1—C7—H7A	108.7	C17—C16—H16	119.5
C8—C7—H7A	108.7	C12—C17—C16	119.3 (2)
O1—C7—H7B	108.7	C12—C17—H17	120.3
C8—C7—H7B	108.7	C16—C17—H17	120.3
H7A—C7—H7B	107.6	C10—N1—N2	115.8 (2)
C9—C8—C10	123.8 (2)	C11—N2—N1	120.31 (19)
C9—C8—C7	117.5 (2)	C11—N2—H2A	119.8
C10—C8—C7	118.4 (2)	N1—N2—H2A	119.8
C8—C9—C5	121.8 (2)	C11—N3—C12	127.5 (2)
C8—C9—Cl1	121.0 (2)	C11—N3—H3A	116.3
C5—C9—Cl1	117.22 (17)	C12—N3—H3A	116.3
N1—C10—C8	119.6 (2)	C6—O1—C7	117.04 (19)
			
C6—C1—C2—C3	−0.3 (4)	C9—C8—C10—N1	179.3 (2)
C1—C2—C3—C4	0.2 (4)	C7—C8—C10—N1	5.2 (3)
C2—C3—C4—C5	0.6 (4)	C17—C12—C13—C14	−0.8 (3)
C3—C4—C5—C6	−1.2 (4)	N3—C12—C13—C14	−176.0 (2)
C3—C4—C5—C9	176.4 (2)	C12—C13—C14—C15	−0.4 (4)
C2—C1—C6—O1	−174.5 (2)	C13—C14—C15—C16	1.1 (4)
C2—C1—C6—C5	−0.3 (4)	C14—C15—C16—C17	−0.7 (4)
C4—C5—C6—O1	175.1 (2)	C13—C12—C17—C16	1.2 (3)
C9—C5—C6—O1	−2.7 (3)	N3—C12—C17—C16	176.3 (2)
C4—C5—C6—C1	1.1 (3)	C15—C16—C17—C12	−0.5 (4)
C9—C5—C6—C1	−176.8 (2)	C8—C10—N1—N2	−176.1 (2)
O1—C7—C8—C9	27.7 (3)	N3—C11—N2—N1	8.2 (3)
O1—C7—C8—C10	−157.8 (2)	S1—C11—N2—N1	−172.62 (17)
C10—C8—C9—C5	−177.1 (2)	C10—N1—N2—C11	−179.3 (2)
C7—C8—C9—C5	−2.9 (3)	N2—C11—N3—C12	−176.3 (2)
C10—C8—C9—Cl1	3.5 (3)	S1—C11—N3—C12	4.7 (4)
C7—C8—C9—Cl1	177.74 (17)	C17—C12—N3—C11	52.3 (3)
C4—C5—C9—C8	172.2 (2)	C13—C12—N3—C11	−132.6 (2)
C6—C5—C9—C8	−10.1 (3)	C1—C6—O1—C7	−157.2 (2)
C4—C5—C9—Cl1	−8.4 (3)	C5—C6—O1—C7	28.6 (3)
C6—C5—C9—Cl1	169.29 (17)	C8—C7—O1—C6	−40.3 (3)

### (II) (E)-2-[(4-Chloro-2H-chromen-3-yl)methylidene]-N-phenylhydrazinecarbothioamide Hydrogen-bond geometry (Å, º)

Cg1 is the centroid of the C12–C17 phenyl ring.

**Table:**

*D*—H···*A*	*D*—H	H···*A*	*D*···*A*	*D*—H···*A*
N2—H2*A*···S1^i^	0.86	2.61	3.456 (2)	167
C2—H2···*Cg*1^ii^	0.93	2.86	3.697 (3)	151

Symmetry codes: (i) −*x*+2, −*y*+2, −*z*; (ii) −*x*+1, −*y*, −*z*.

## References

[bb1] Antholine, W., Knight, J., Whelan, H. & Petering, D. H. (1977). *Mol. Pharmacol.* **13**, 89–98. 834188

[bb2] Beraldo, H. & Gambino, D. (2004). *Mini. Rev. Med. Chem.* **4**, 31–39. 10.2174/1389557043487484 14754441

[bb3] Bruker (2008). *APEX2*, *SAINT* and *SADABS*. Bruker AXS Inc., Madison, Wisconsin, USA.

[bb4] Farrugia, L. J. (2012). *J. Appl. Cryst.* **45**, 849–854.

[bb5] Finch, R. A., Liu, M. C., Cory, A. H., Cory, J. G. & Sartorelli, A. C. (1999). *Adv. Enzyme Regul.* **39**, 3–12. 10.1016/s0065-2571(98)00017-x 10470363

[bb6] Gangadharan, R., Haribabu, J., Karvembu, R. & Sethusankar, K. (2014). *Acta Cryst.* E**70**, o1039–o1040. 10.1107/S1600536814018509 PMC4186115 25309214

[bb7] Groom, C. R. & Allen, F. H. (2014). *Angew. Chem. Int. Ed.* **53**, 662–671. 10.1002/anie.201306438 24382699

[bb8] Ilies, D.-C., Pahontu, E., Shova, S., Georgescu, R., Stanica, N., Olar, R., Gulea, A. & Rosu, T. (2014). *Polyhedron*, **81**, 123–131.

[bb9] Joseph, M., Suni, V., Nayar, C. R., Kurup, M. R. P. & Fun, H.-K. (2004). *J. Mol. Struct.* **705**, 63–70.

[bb10] Kumbhar, A., Sonawane, P., Padhye, S., West, D. X. & Butcher, R. J. (1997). *J. Chem. Crystallogr.* **27**, 533–539.

[bb12] Kurup, M. R. P. & Joseph, M. (2003). *Synth. React Inorg. Met. Org. Chem.* **33**, 1275–1287.

[bb11] Macrae, C. F., Bruno, I. J., Chisholm, J. A., Edgington, P. R., McCabe, P., Pidcock, E., Rodriguez-Monge, L., Taylor, R., van de Streek, J. & Wood, P. A. (2008). *J. Appl. Cryst.* **41**, 466–470.

[bb13] Sartorelli, A. C., Moore, E. C., Zedeck, M. S. & Agrawal, K. C. (1970). *Biochemistry*, **9**, 4492–4498. 10.1021/bi00825a005 5529812

[bb14] Sheldrick, G. M. (2008). *Acta Cryst.* A**64**, 112–122. 10.1107/S0108767307043930 18156677

[bb15] Spek, A. L. (2009). *Acta Cryst.* D**65**, 148–155. 10.1107/S090744490804362X PMC2631630 19171970

[bb16] Thelander, L. & Gräslund, A. (1983). *J. Biol. Chem.* **258**, 4063–4066. 6300073

[bb17] Vimala, G., Govindaraj, J., Haribabu, J., Karvembu, R. & SubbiahPandi, A. (2014). *Acta Cryst.* E**70**, o1151. 10.1107/S1600536814021667 PMC4257262 25484795

